# Low‐Coordination Configuration Single‐Atom Manganese Nanozymes for NIR‐Imaging‐Oriented Efficient Catalytic Oncotherapy

**DOI:** 10.1002/advs.202502664

**Published:** 2025-03-17

**Authors:** Peiwei Jin, Dandan Wang, Yijun Lu, Yong Qian, Chengyang Fang, Xiaoxiao Zhang, Junchao Qian, Songnan Qu, Hui Wang

**Affiliations:** ^1^ University of Science and Technology of China Hefei Anhui 230026 P. R. China; ^2^ High Magnetic Field Laboratory Hefei Institutes of Physical Science Chinese Academy of Science Hefei Anhui 230031 P. R. China; ^3^ Institute of Health and Medical Technology Hefei Institutes of Physical Science Chinese Academy of Sciences Hefei Anhui 230031 P. R. China; ^4^ Joint Key Laboratory of the Ministry of Education Institute of Applied Physics and Materials Engineering University of Macau Taipa Macau SAR 999078 P. R. China

**Keywords:** carbon dots, catalytic effect, Mn‐N_2_, NIR‐imaging, single atom nanozyme

## Abstract

Single‐atom nanozymes (SAzymes) with low coordination structure has more active sites at an atomic level, which is one of the effective strategies to improve the efficiency of tumor catalytic therapy. Herein, it is reported that simple preparation of single‐atom manganese‐doped carbon dots (SA Mn‐CDs) with low coordination (Mn‐N_2_) configuration formed by chelating manganese atoms with ethylenediamine tetraacetic acid (EDTA). SA Mn‐CDs show good dispersibility of different solvents, excellent biological safety and stability, and show deep level (34 µm) of two‐photon fluorescence imaging ability and exclusive function (Pearson's correlation coefficient: 0.752) of targeting lysosomes at the cellular level. This further promotes the high‐efficiency peroxidase (POD)‐like activity (SA = 0.64 U mg^−1^) produced by the Fenton‐like reaction mediated by SA Mn‐CDs and shows excellent ability to kill tumor cells and shrink tumor tissues in vitro and in vivo respectively. This work proves the great potential of SA Mn‐CDs nanozyme in near‐infrared fluorescence‐guided tumor catalytic therapy.

## Graphical abstract

The manuscript designs a type of SA Mn‐CDs with low coordination Mn‐N_2_ configuration hydrothermally synthesized by chelating manganese atoms with ethylenediamine tetraacetic acid. SA Mn‐CDs not only have more active sites at the atomic level for high efficiently peroxidase‐like activity but also show excellent fluorescent ability at the cellular and biological levels for near‐infrared imaging guided tumor catalytic therapy.

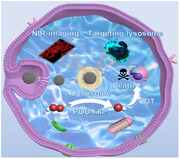

## 1 Introduction

In recent years, single‐atom nanozymes (SAzymes) have been widely concerned in the fields of energy catalysis, life science, and nano‐medicine, because of their well‐designed coordination structure, which endows them with extraordinary catalytic ability.^[^ ^1^ ^]^ Currently, most reported metal single atoms based on nitrogen coordination (M‐N_x_) exist in a stable M‐N_4_ configuration, and the active sites exposed by the central metal atom show significantly higher catalytic activity than nanoparticles.^[^ ^2^ ^]^ However, the limited number of active sites depends too much on the sensitivity of substrate adsorption, which leads to the easy separation of intermediates and reduces the catalytic efficiency.^[^ ^3^ ^]^ It is a promising method to improve the catalytic efficiency of SAzymes by reducing the coordination number of metal atoms and providing more unoccupied atomic orbitals.^[^ ^4^ ^]^ Therefore, it is crucial to choose suitable carriers to stabilize the configuration of metal atoms in low coordination structures.

Riveting SAzymes in carbon‐based materials is one of the popular methods to stabilize monatomic configuration.^[^ ^5^ ^]^ Carbon‐based quantum dots with unique luminescent characteristics, good stability, and tunable physicochemical properties are one of the effective candidate materials as carriers.^[^ ^6^ ^]^ Combined with the carbon dots (CDs) of SAzymes, it not only has good multi‐modal imaging ability but also can visually realize the imaging‐oriented therapeutic function in vitro and in vivo.^[^ ^7^ ^]^ It is also possible to achieve efficient anchoring of central metal atoms and sufficient exposure of catalytic active sites through abundant dangling bonds (─OH, ─C═O, and ─NH_2_) on the surface of CDs.^[^ ^8^ ^]^ However, conventional methods (such as the thermal reduction method) for preparing monoatomic catalysts are difficult to achieve precise regulation of the coordination environment.^[^ ^9^ ^]^ Therefore, choosing appropriate ligands to chelate the central metal atoms to form a stable low‐coordination topological structure is the premise of constructing SAzymes with low‐coordination structure.^[^ ^10^ ^]^

**Scheme 1:**
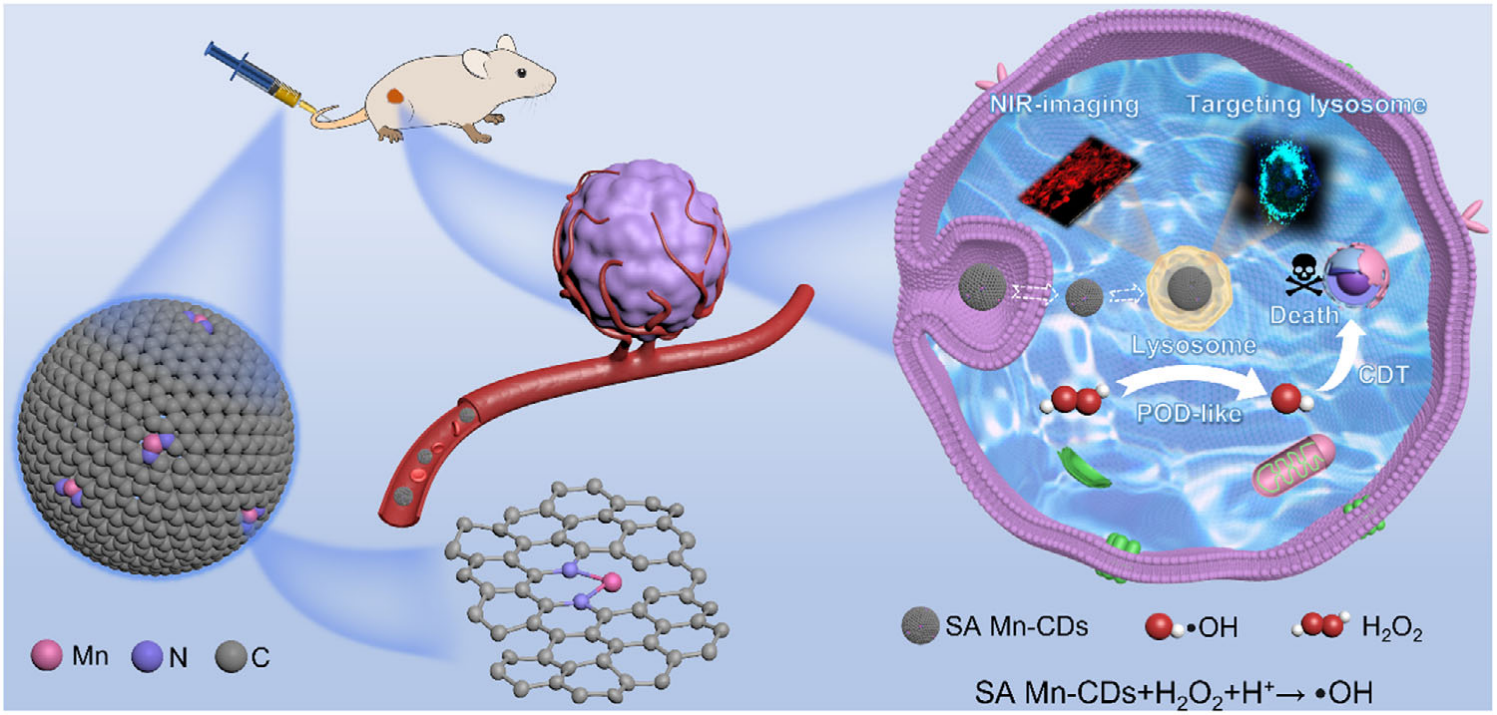
Schematic diagram of SA Mn‐CDs for NIR‐imaging‐oriented efficient catalytic oncotherapy.

Herein, we chelated manganese with ethylenediamine tetraacetic acid (EDTA) as a precursor, and synthesized single‐atom manganese‐doped carbon dots (SA Mn‐CDs) with low coordination Mn‐N_2_ configuration by one‐pot hydrothermal method. Ultra‐small‐sized SA Mn‐CDs with the atomically dispersed manganese center, showing good water solubility and biological safety, and endowing it with excellent peroxidase (POD)‐like activity. SA Mn‐CDs with CDs fluorescence characteristics can also show excellent imaging ability at the cellular and biological levels, and realize the ability of near‐infrared fluorescence imaging guided lysosomal targeted tumor catalytic therapy (**Scheme** **1**). This work further emphasizes the critical role of structural design in the context of single‐atom catalytic therapy.

## 2 Results and Discussion

### 2.1 Synthesis and Characterizations of the SA Mn‐CDs

**Figure 1:**
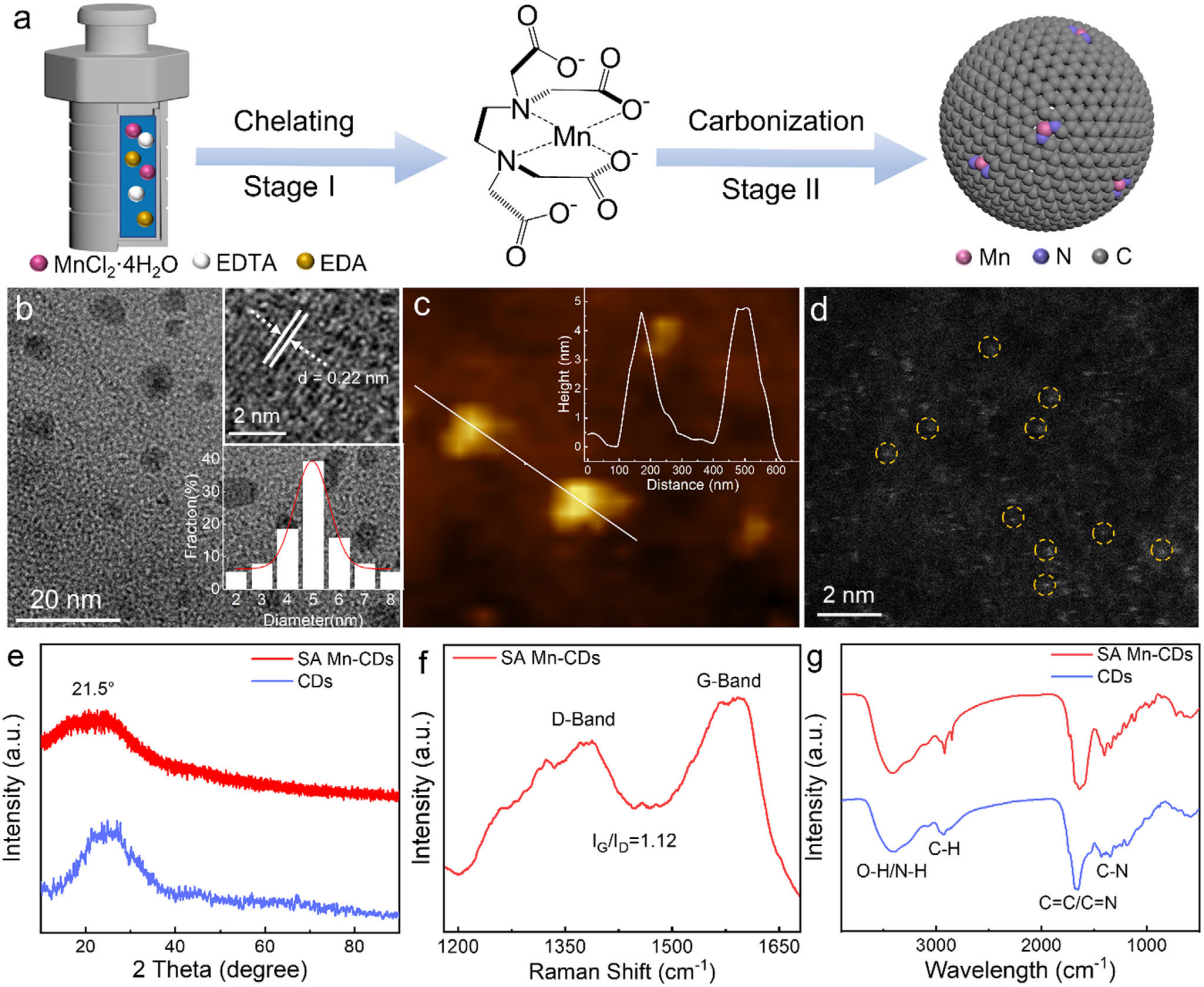
Synthetic process and characterization of SA Mn‐CDs. a) Schematic diagram of the synthesis of SA Mn‐CDs. b) TEM image of SA Mn‐CDs. The insets are the lattice fringe (top right) and size distribution (bottom right) of SA Mn‐CDs. c) AFM image and height profile of SA Mn‐CDs. d) HAADF‐STEM image of SA Mn‐CDs. e) XRD pattern of SA Mn‐CDs and CDs. f) Raman spectrum of SA Mn‐CDs and g) FT‐IR spectrum of SA Mn‐CDs and CDs.

To accurately control the preparation of SA Mn‐CDs with low coordination configuration, it is necessary to chelate the central Mn atoms with different ligands to form a stable topological precursor, and EDTA is the main chelating agent here. As shown in **Figure** **1**a, first, the Mn source was mixed with EDTA aqueous solution ultrasonically, and the carboxyl and amino groups in EDTA dissociated into hydrogen ions, and then formed ligand bonds to chelate with Mn to obtain a stable complex structure; Second, because the dehydrogenated carboxyl and amino groups had different energies to form ligand bonds with Mn, the manganese–oxygen ligand bonds were preferentially broken by controlling the reaction conditions, and then only the chelating structure of manganese–nitrogen bonds were left; Finally, under the reaction conditions of high temperature and high pressure, the precursor of this chelating structure with manganese–nitrogen bonds continued to degrade and carbonize, and SA Mn‐CDs with low coordination structure was formed.

The physical properties of SA Mn‐CDs were characterized. As shown in Figure 1b, the transmission electron microscopy (TEM) image showed that the obtained SA Mn‐CDs was uniformly dispersed and the size distribution was within the range of 5 ± 3 nm (inset in Figure 1b, lower right), and the high‐resolution transmission electron microscopy (HRTEM) image revealed that the crystal plane spacing of 0.22 nm corresponds to the (100) crystal plane of graphite carbon (inset in Figure 1b, upper right).^[^ ^11^ ^]^ The size of the SA Mn‐CDs was further confirmed by atomic force microscopy (AFM) image, showing that its height was ≈5 nm (Figure 1c and the illustration). Pure CDs exhibited dimensions analogous to the SA Mn‐CDs (Figure S1, Supporting Information). Atomic resolution high‐angle annular dark field scanning transmission electron microscopy (HAADF‐STEM) with aberration correction was employed to detect the presence of monatomic manganese. As shown in Figure 1d, the SA Mn were identified as isolated bright spots, indicating that successfully loaded on the carbon carrier.

The disordered core structure of SA Mn‐CDs was confirmed by X‐ray diffraction (XRD). The diffraction bands of SA Mn‐CDs are centered at 21.5°, which suggests that the sp^2^ structural domains in their cores lack an ordered *π*–*π* stacking (Figure 1e). SA Mn‐CDs exhibited a leftward shift of ≈1° in comparison to CDs, which might be attributable to the effective doping of manganese. The Raman spectrum of SA Mn‐CDs showed the characteristic peaks at 1588 and 1370 cm^−1^, which corresponded to the G band and D band respectively, and the ratio of them was 1.12, suggesting the presence of a low density of vacancy defects (Figure 1f). The SA Mn‐CDs were subjected to further analysis via Fourier transform‐infrared (FT‐IR) spectroscopy. As illustrated in Figure 1g, the FT‐IR spectrum of SA Mn‐CDs and CDs in the broad absorption band of 3400 cm^−1^ was attributed to the O─H/N─H stretching vibration. Moreover, the remaining functional groups were identified as C─N at 1402 cm⁻^1^, C═C/C═N at 1637 cm⁻^1,^ and C─H at 2919 cm⁻^1^, respectively.^[^ ^12^ ^]^ The presence of copious hydrophilic groups and negative potential (−21.89 mV) on the surface of SA Mn‐CDs (Figure S2, Supporting Information), which endows it with excellent stability in various solvents such as aqueous solution (Figure S3, Supporting Information) and cell culture medium (Figure S4, Supporting Information).

**Figure 2:**
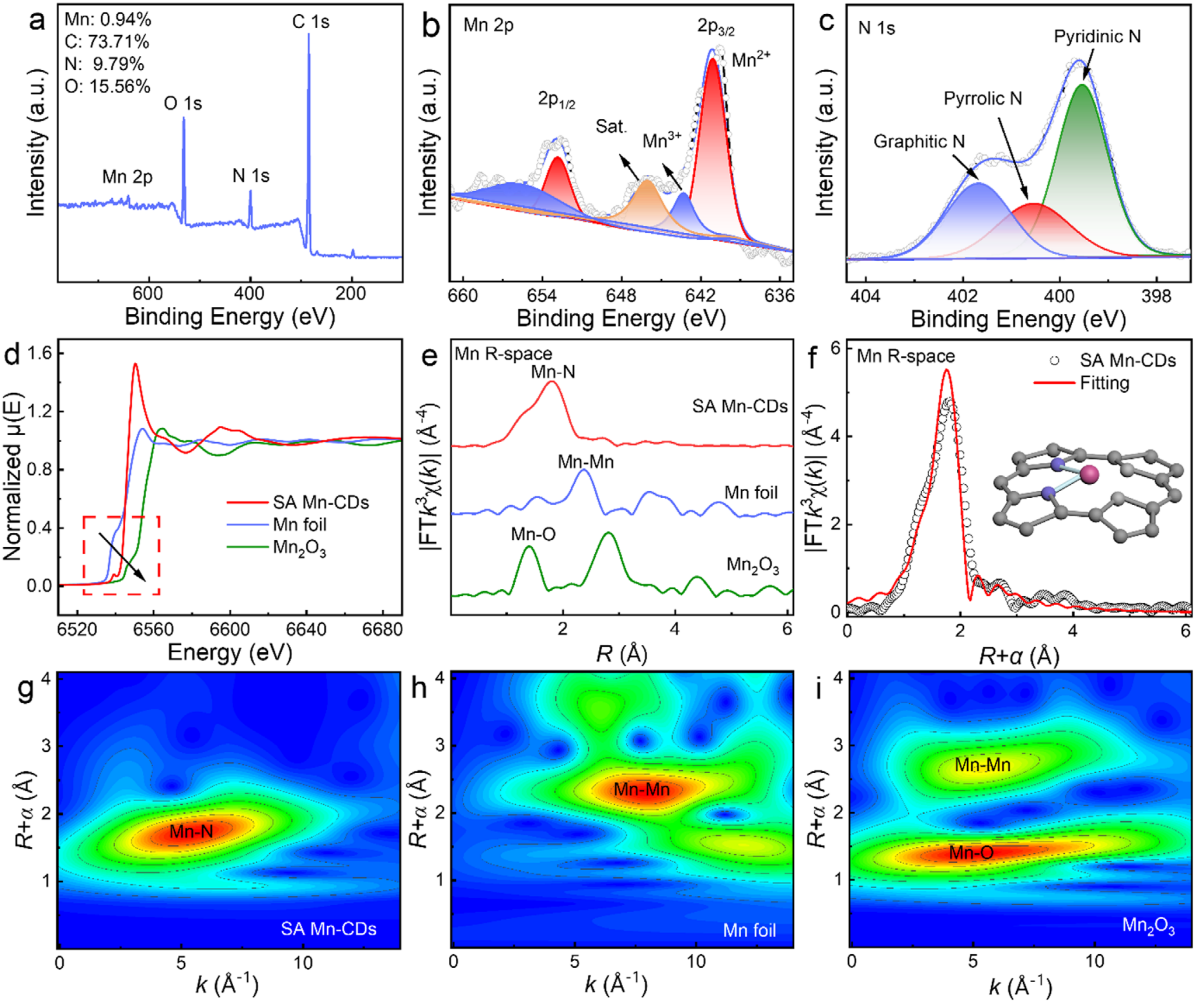
Structural characterization of SA Mn‐CDs. a) Survey XPS spectrum of the SA Mn‐CDs. b,c) High‐resolution XPS spectra of Mn 2p and N 1s, respectively. d) The XANES spectra and e) FT‐EXAFS R‐space curves of SA Mn‐CDs and reference samples (Mn foil and Mn_2_O_3_). f) FT‐EXAFS fitting curves at R space of Mn K‐edge for SA Mn‐CDs (inset, schematic model of local structure of MnN_2_). WT‐EXAFS plots of g) SA Mn‐CDs, h) Mn foil and (i) Mn_2_O_3_.

The surface chemistry of the SA Mn‐CDs was subsequently examined through the application of X‐ray photoelectron spectroscopy (XPS). As shown in **Figure** **2**a, the surface spectrum of SA Mn‐CDs showed that there were Mn, C, N, and O elements, and the atomic percentages (at%) of the corresponding elements were 0.94%, 73.71%, 9.79%, and 15.56%, respectively. There were two main peaks at 653.1 and 641.1 eV and a characteristic peak at 645.9 eV in the high‐resolution Mn 2p spectrum of SA Mn‐CDs, which belong to Mn 2p_1/2_, Mn 2p_3/2_ and satellite peak, respectively (Figure 2b). Two peaks at 641.3 and 652.1 eV were identified from the Mn^2+^ peak, while the other two peak at 643 and 655.2 eV were associated with Mn^3+^, which suggested that the elemental Mn is co‐existing as Mn^2+^ and Mn^3+^ in SA Mn‐CDs.^[^ ^13^ ^]^ The N 1s peak of SA Mn‐CDs can be deconvoluted into three peaks of 399.4, 400.6, and 401.7 eV, corresponding to pyridine‐N, pyrrolic‐N, and graphite‐N, respectively (Figure 2c).^[^ ^14^ ^]^ Additionally, the C 1s high‐resolution XPS spectra of SA Mn‐CDs exhibited a high degree of correlation with the C─C/C═C, C─O/C─N, C═N, and C═O peaks at 284.8, 285.9, 287.4, and 288.2 eV, respectively (Figure S5, Supporting Information).^[^ ^15^ ^]^ In parallel, the O 1s XPS spectra of SA Mn‐CDs exhibited three distinct peaks at 531.1 and 532.1 eV binding energies, which corresponded to lattice oxygen and adsorbed oxygen, respectively (Figure S6, Supporting Information).^[^ ^16^ ^]^

X‐ray absorption near‐edge structure (XANES) spectra were employed to determine the valence information of Mn in SA Mn‐CDs. As illustrated in Figure 2d, the XANES spectra demonstrated that the adsorption peak position of SA Mn‐CDs was situated between the adsorption peak positions of Mn foil and Mn_2_O_3_, thereby further substantiating its characteristic Mn^δ+^ (0 <δ <3) electronic structure, which was in accordance with the preceding XPS analysis. Fourier transform extended X‐ray absorption fine structure (FT‐EXAFS) characterization was used to further verify the bonding type in the SA Mn‐CDs. As shown in Figure 2e, the main peak of single Mn foil at 2.33 Å was attributed to Mn─Mn coordination, while the FT‐EXAFS spectrum of Mn_2_O_3_ had obvious characteristic peaks at 1.41 Å and 2.81 Å, corresponding to Mn─O and Mn─Mn coordination, respectively. Therefore, the main peak of the EXAFS spectrum of SA Mn‐CDs at 1.78 Å was attributed to Mn─N coordination. The absence of the Mn─Mn bond peak in the SA Mn‐CDs sample indicated the absence of Mn clusters and the atomic distribution of the Mn─N site. The structural parameters of SA Mn‐CDs were obtained through quantitative least‐squares EXAFS curve fitting in K‐space (Figure S7, Supporting Information). The result of the curve fitting was in good agreement with the experimental data.

To accurately ascertain the coordination structure of Mn, a quantitative EXAFS curve fitting in R‐space on the Mn *k*‐edge was undertaken to derive the structural parameters of Mn. Figure 2f shows the best fitting for SA Mn‐CDs. Meanwhile, the EXAFS spectra of Mn foil and Mn_2_O_3_ were subjected to analysis and fitting in R‐space and K‐space, respectively (Figures S8 and S9, Supporting Information). The average Mn─N coordination number of SA Mn‐CDs was calculated to be 2.2 based on the EXAFS fitting parameters (Table S1, Supporting Information), indicating that the Mn atoms in the SA Mn‐CDs structure were coordinated with two N atoms. Furthermore, the wavelet transforming (WT) of the EXAFS oscillations offered a more pronounced resolution of the localized structure in both K and R spaces and further analyzed the atomic configurations. The WT isogram of SA Mn‐CDs showed the single intensity maximum (5.4 Å^−1^) corresponding to Mn─N coordination in R and K spaces (Figure 2g). The WT isograms of a single Mn foil and Mn_2_O_3_ showed the maximum intensity values of the scattering paths corresponding to Mn─O and Mn─Mn, respectively. The aforementioned results illustrated that the atomically dispersed Mn in SA Mn‐CDs exists in the Mn‐N_2_ configuration.

### 2.2 Optical Properties of SA Mn‐CDs

**Figure 3:**
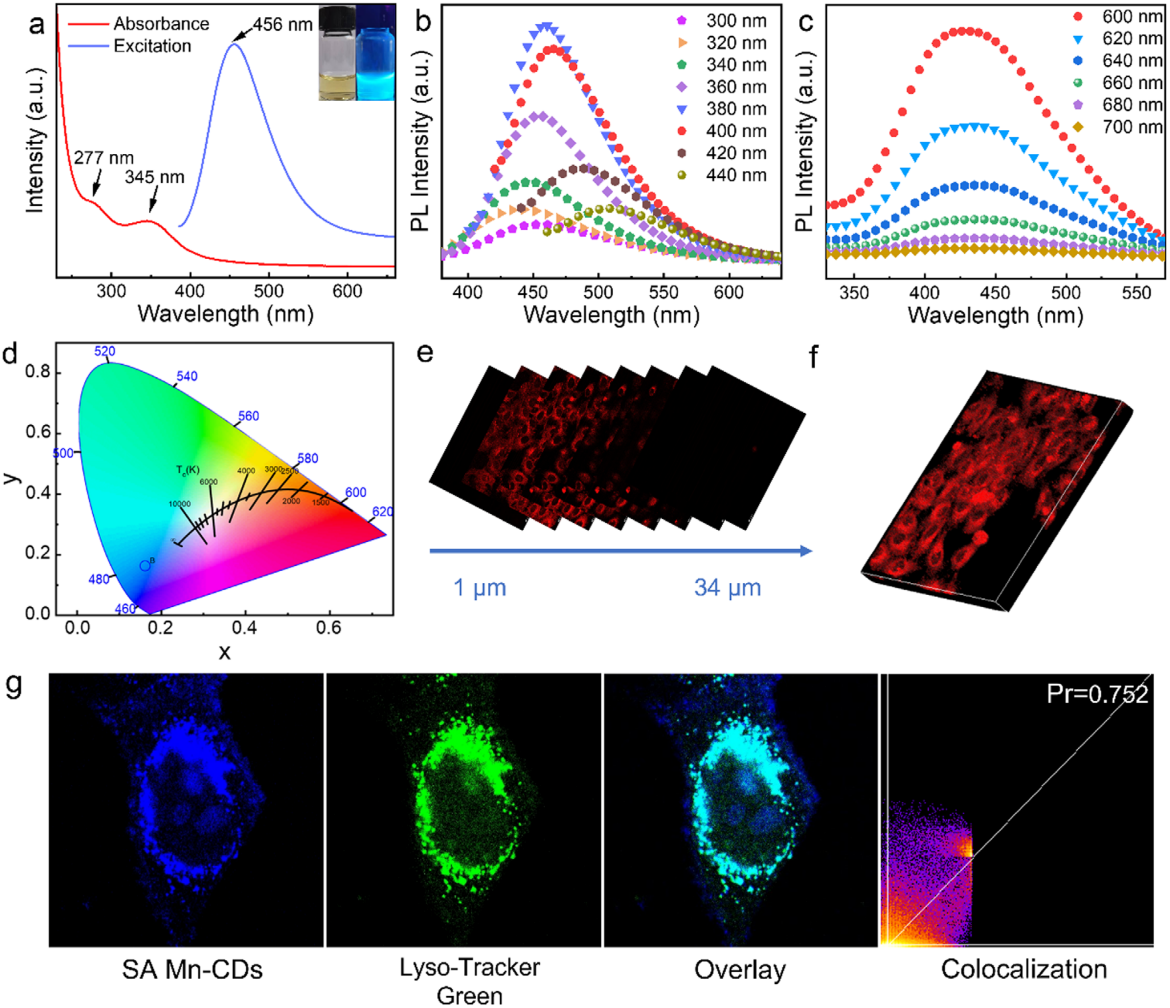
Optical properties of SA Mn‐CDs. a) The absorbance and excitation spectra of SA Mn‐CDs. The inset is the photograph of SA Mn‐CDs taken in daylight (left) and UV light (right) with a wavelength of 365 nm. b)Down‐converted and c) up‐converted fluorescence emission of SA Mn‐CDs. d) CIE coordinates of SA Mn‐CDs. e) The two‐photon fluorescent images of fixed tumor cells stained with SA Mn‐CDs at different penetration depths along the *z*‐axis and f) reconstructed 3D fluorescent images. g) Two‐photon colocalization imaging of tumor cells stained with SA Mn‐CDs and Lyso‐Tracker Green dye.

SA Mn‐CDs supported on carbon dots have the potential of fluorescence imaging to further characterize its optical characteristics. As shown in **Figure** **3**a, the UV–vis absorption spectrum showed distinct absorption peaks at 277 and 345 nm, which corresponded to *π*→*π* ^*^ transition of aromatic C═C bond and n→*π* ^*^ transition of aromatic sp^2^ system containing C═O bond, respectively. The maximum fluorescence intensity was near 456 nm at an excitation wavelength of 380 nm. Furthermore, the synthesized SA Mn‐CDs aqueous solution was light yellow and emitted blue fluorescence under UV light irradiation (inset in Figure 3a). As shown in Figure 3b, the fluorescence peak of SA Mn‐CDs shifted from 440 to 530 nm as the excitation wavelength increased from 300 to 440 nm, accompanied by a redshift of the fluorescence emission position. The SA Mn‐CDs exhibited up‐converted fluorescence emission from 425 to 450 nm under longer wavelength excitation (600–700 nm) (Figure 3c). In addition, the fluorescence intensity of SA Mn‐CDs decayed by only 2.49% after 365 nm laser irradiation for 1 h, indicating that SA Mn‐CDs had good photostability (Figure S10, Supporting Information). The Commission Internationale de L'Eclairage (CIE) coordinates of SA Mn‐CDs in Figure 3d were (0.16, 0.16), and the color of fluorescence should theoretically be blue, which corresponded to the actual results.

4T1 cells were used as a model to evaluate the in vitro bioimaging capability of SA Mn‐CDs. SA Mn‐CDs produced bright fluorescent signals when 4T1 cells were irradiated at 405, 488, 561, and 640 nm (Figure S11, Supporting Information). Moreover, flow cytometry revealed that SA Mn‐CDs could be rapidly internalized by cells (Figure S12, Supporting Information). In vitro two‐photon imaging of 4T1 cells incubated with SA Mn‐CDs using a pulsed near‐infrared laser at a wavelength of 760 nm. As shown in Figure 3e,f, the fluorescence images along the *z*‐axis direction at different penetration depths and the reconstructed 3D fluorescence images showed that the maximum fluorescence image depth of two‐photon can reach 34 µm. Compared with the single‐photon fluorescence images, it presented a deeper penetration depth (Figure S13, Supporting Information). Co‐localization experiments were performed to verify the ability of SA Mn‐CDs to selectively accumulate in lysosomes of living cells. The blue fluorescence of SA Mn‐CDs overlapped well with the green fluorescence of commercial lysosomal probe Lyso–Tracker Green, and Pearson's correlation coefficient was calculated as 0.752 (Figure 3g).^[^ ^17^ ^]^ The above results showed that SA Mn‐CDs had excellent lysosomal targeting ability, which was beneficial to the release of acidic substances in lysosomes, and thus enhanced the chemodynamic therapy (CDT) ability.^[^ ^18^ ^]^

### 2.3 Catalytic properties of SA Mn‐CDs in vitro

**Figure 4:**
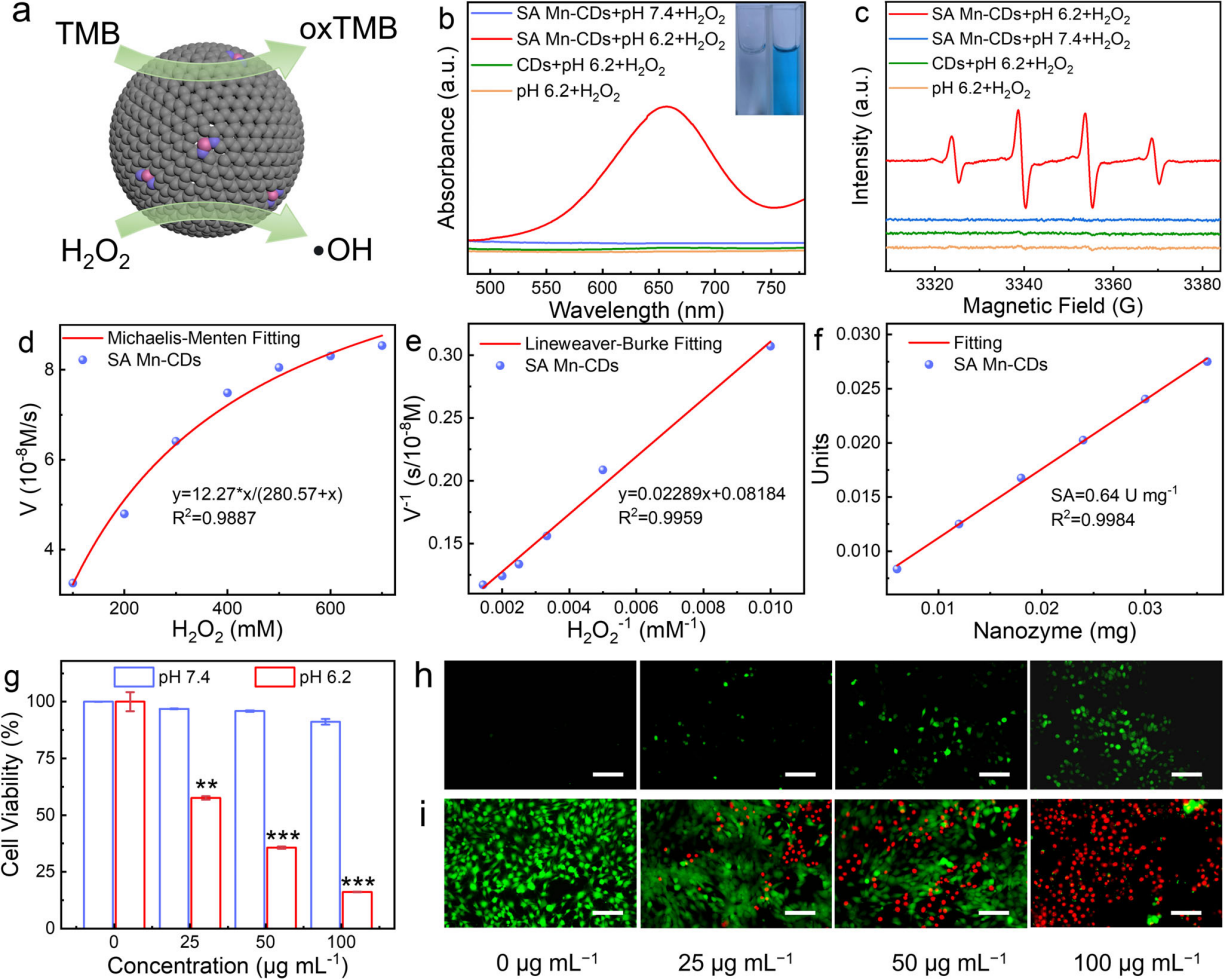
Catalytic properties of SA Mn‐CDs. a) Schematic illustration of POD‐like function of SA Mn‐CDs. b) TMB colorimetric reaction and c) detection of •OH by EPR of SA Mn‐CDs and CDs under different pH conditions. Michaelis‐Menten fitting curve d) and Lineweaver‐Burke fitting curve e) of •OH generation rate as a function of H_2_O_2_ concentration in the presence of SA Mn‐CDs and TMB. f) The specific activities of SA Mn‐CDs. g) Relative viabilities of 4T1 cells treated with different concentrations of SA Mn‐CDs. h) ROS staining and i) Dead/live staining of SA Mn‐CDs‐treated 4T1 cells with different concentrations at pH 6.2. The scale bar is 200 µm. Statistical analysis was performed using the unpaired Student's t‐test (^**^ *p* < 0.01, ^***^ *p* < 0.001, ^****^ *p* < 0.0001).

The POD‐like activity of the prepared SA Mn‐CDs was evaluated by the 3,3′,5,5′‐tetramethylbenzidine (TMB) chromogenic assay (**Figure** **4**a). As shown in Figure 4b, only in the acidic conditions, the color of the TMB solution coexisting with SA Mn‐CDs turned blue obviously, and a distinct absorption peak appeared at 652 nm of UV–vis absorption spectrum. With 5,5‐dimethyl ‐1‐ pyrroline N‐ oxide (DMPO) as scavenger and electron paramagnetic resonance (EPR) spectrum, the ability of SA Mn‐CDs to produce hydroxyl radical (•OH) was further evaluated. The acidic solution with SA Mn‐CDs and H_2_O_2_ solution showed a clear characteristic signal (1:2:2:1) of •OH (Figure 4c). The change in absorbance of the reaction solution with time was monitored by UV–vis spectroscopy at 652 nm. As shown in Figures S14 and S15 (Supporting Information), the absorbance gradually increased with increasing concentration of H_2_O_2_ or TMB. The Michaelis–Menten steady‐state kinetics study was used to determine the catalytic efficiency of SA Mn‐CDs. When H_2_O_2_ with different concentrations was used as a substrate, the maximum reaction rate (V_max_) and Michaelis constant (K_m_) of SA Mn‐CDs were ≈0.12 µM s^−1^ and 0.28 mm respectively (Figure 4d,e).^[^ ^19^ ^]^ However, when TMB with different concentrations was used as a substrate, the V_max_ and K_m_ of SA Mn‐CDs were ≈0.34 × 10^−7^ m s^−1^ and 0.49 mm (Figures S16 and S17, Supporting Information), indicating that SA Mn‐CDs was more inclined to use H_2_O_2_ as a substrate to obtain high catalytic rate. Furthermore, the specific activity (SA, U mg^−1^) was used to quantify the nano‐enzyme activity of SA Mn‐CDs. As shown in Figure 4f, the SA value of SA Mn‐CDs reached 0.64 U mg^−1^, indicating that it had good POD‐like activity.

4T1 cell line was used to evaluate in vitro anti‐tumor activity of SA Mn‐CDs. 4T1 cells were treated with different concentrations of SA Mn‐CDs under neutral or acidic conditions, and cell activity was assessed by the CCK‐8 assay. Under neutral conditions, the cells remained highly viable even if the concentration of SA Mn‐CDs increased to 100 µg mL^−1^. In contrast, the cell viability decreased significantly (16.5%) with the same concentration of SA Mn‐CDs under acidic conditions (pH 6.2) (Figure 4g). The above results suggested that the acidic condition was necessary for SA Mn‐CDs to achieve anti‐tumor activity. 2,7‐Dichlorodihydrofluorescein (DCFH‐DA) was used to evaluate intracellular •OH production in 4T1 cells treated with SA Mn‐CDs. As illustrated in Figure 4h and Figure S18 (Supporting Information), the intensity of green fluorescence associated with intracellular ROS levels increased in a sequential manner with rising concentrations (0, 25, 50, and 100 µg mL^−1^) of SA Mn‐CDs, indicating that SA Mn‐CDs entered cells under acidic condition and catalyzed the generation of •OH from H_2_O_2_ via POD‐like activity. However, there was no significant change in fluorescence intensity with various concentrations of pH 7.4 (Figure S19, Supporting Information). Furthermore, calcein‐AM and propidium iodide (PI) were used to stain the cells treated with SA Mn‐CDs to distinguish the living from dead cells. Under acidic conditions, the red fluorescence intensity produced by SA Mn‐CDs treatment was obviously higher than that of the control group, and the red fluorescence intensity was positively correlated with the concentration of SA Mn‐CDs (Figure 4i; Figure S20, Supporting Information).

### 2.4 In Vivo Biocompatibility

**Figure 5:**
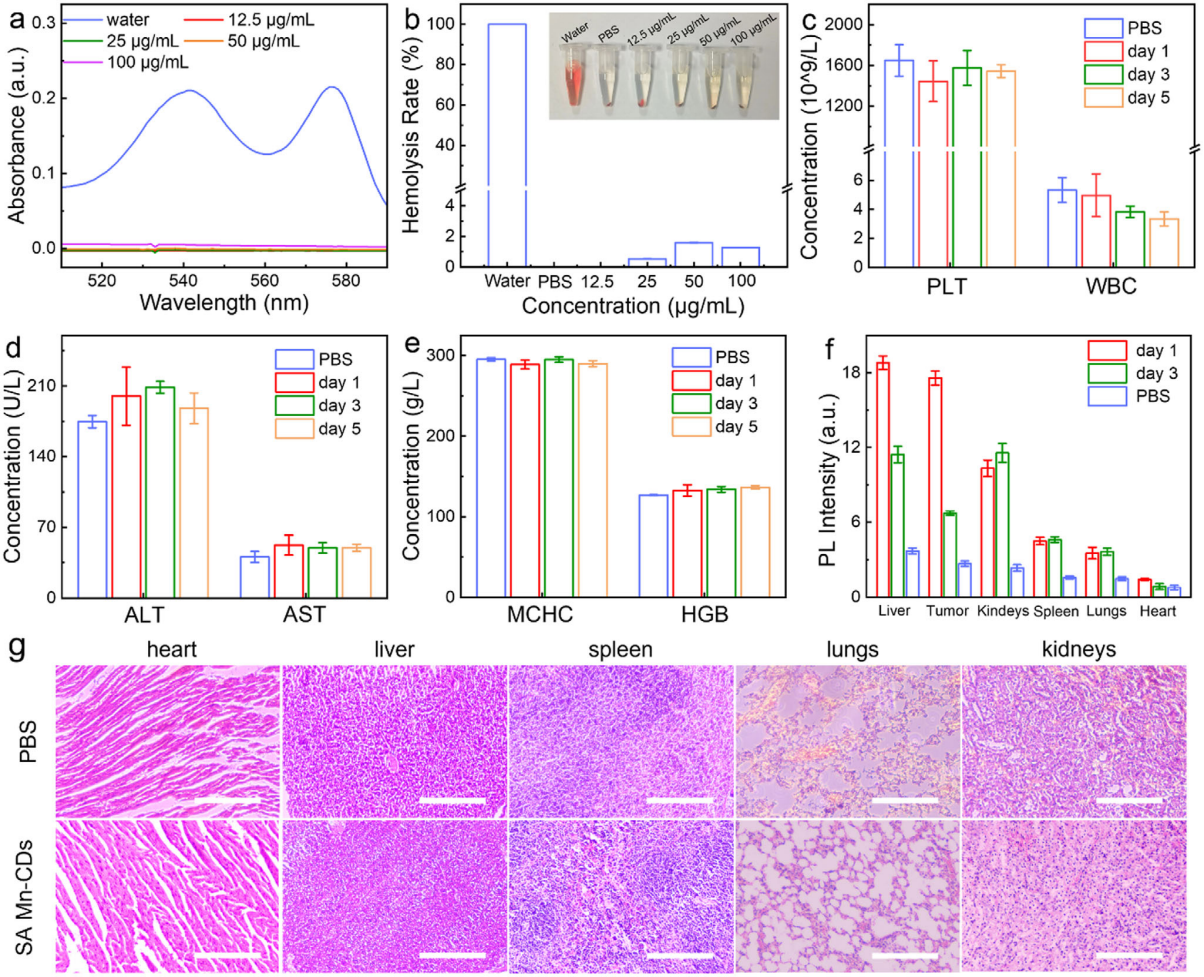
Biosafety assessment of SA Mn‐CDs. a) UV–vis spectra of supernatant solutions of red blood cells incubated with SA Mn‐CDs. b) Hemolysis test of mouse blood exposed to SA Mn‐CDs (n  = 3). The inset image shows the samples after centrifugation. c) PLT and WBC counts of mice treated with SA Mn‐CDs for 24, 72, and 120 h (n = 3). d) ALT and AST counts of mice treated with the SA Mn‐CDs for 24, 72, and 120 h (n = 3). e) MCGC and HGB counts of mice treated with the SA Mn‐CDs for 24, 72, and 120 h (n = 3). f) Biodistribution in major organs and tumor tissues of mice intravenously injected PBS/SA Mn‐CDs for different times (n = 3). g) H&E stained tissue sections of the main organs of mice intravenously injected PBS/SA Mn‐CDs for 5 days. The scale bar is 100 µm.

The biocompatibility and biodistribution of SA Mn‐CDs were assessed by analyzing the blood and organs of nude mice injected with SA Mn‐CDs. The nude mice treated with phosphate‐buffered saline (PBS) were used as the control group. The solubility of SA Mn‐CDs in blood was determined by using UV–vis spectroscopy at 540 nm. As shown in **Figure** **5**a,b, a negligible number of hemolyzed erythrocytes were observed even at the concentration of 100 µg mL^−1^, indicating reasonable biocompatibility. The biological safety of SA Mn‐CDs at the blood level was further detected by blood routine and blood biochemical indexes. There were no notable alterations in aspartate aminotransferase (AST), alanine aminotransferase (ALT), platelet (PLT), white blood cell (WBC), hemoglobin (HGB) and mean corpuscular hemoglobin concentration (MCHC) in the mice following a five‐day of administration, indicating that SA Mn‐CDs demonstrated no hematotoxic effects (Figure 5c–e).

In order to conduct a biodistribution study, mice were executed on days 1 and 3 post‐injection, and the main organs were collected. The biodistribution of SA Mn‐CDs was assessed by quantifying the fluorescence intensity in organs. As shown in Figure 5f and Figure S21 (Supporting Information), the majority of SA Mn‐CDs were concentrated in the liver, followed by the tumor, spleen, and kidney. As anticipated, minimal accumulation was observed in the lungs and heart. The peak accumulation of SA Mn‐CDs in tumor tissues was observed on day 1 post‐injection, followed by a decline. A histological analysis was conducted on the major organs of mice that had been treated with PBS and SA Mn‐CDs. As illustrated in Figure 5g, no discernible abnormalities were observed in the main organs. The aforementioned results indicated that SA Mn‐CDs possessed favorable biocompatibility for in vivo therapeutic applications.

### 2.5 In Vivo Therapy

**Figure 6:**
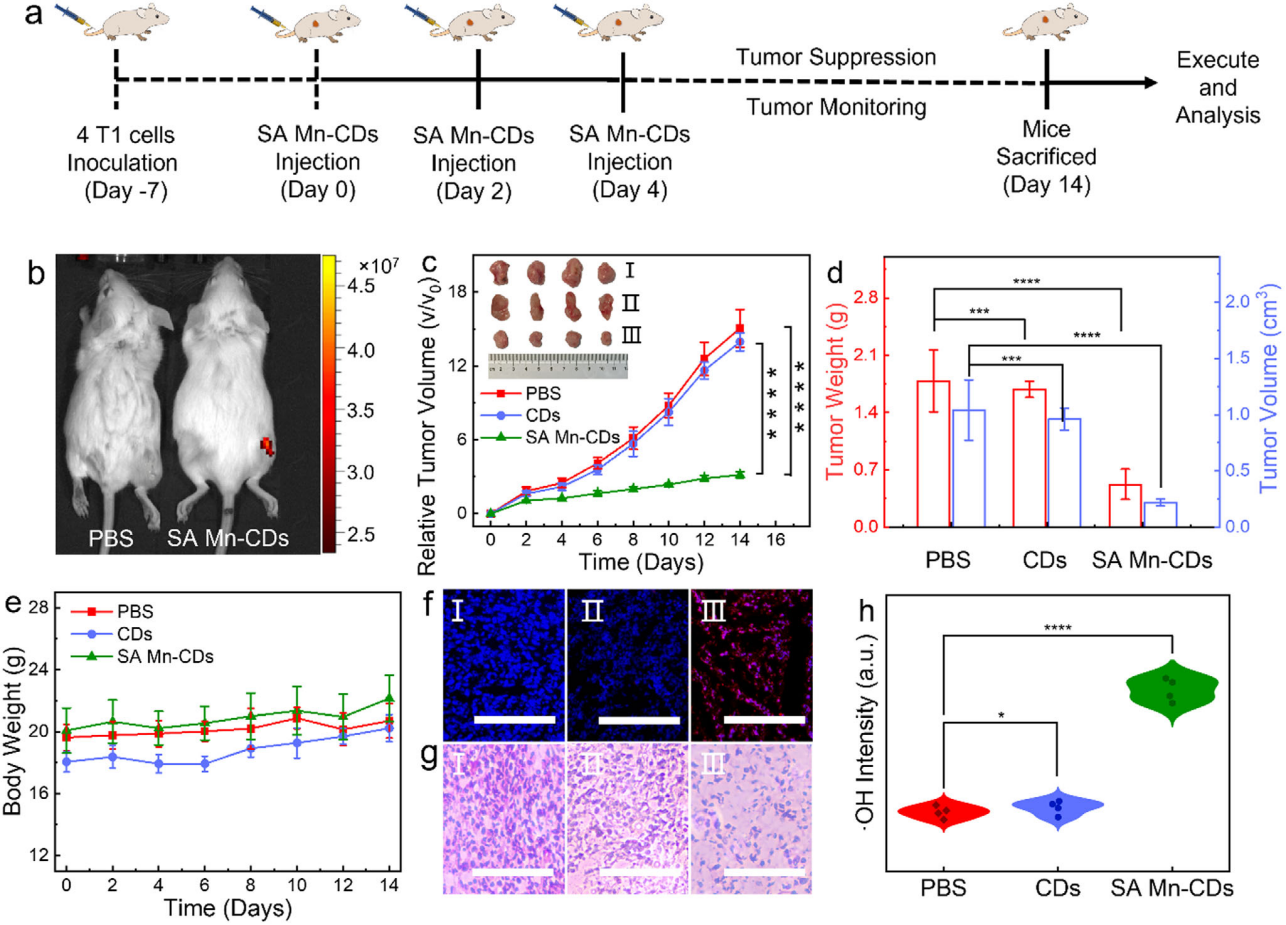
In vivo tumor catalytic therapy of SA Mn‐CDs. a) Schematic diagram of treatment schedule. b) Fluorescence imaging of tumor‐bearing mice intravenously inoculated with SA Mn‐CDs under 680 nm excitation. c) Tumor growth curves of mice under various treatments for a period of 14 days after receiving the first dose of administration. The inset showed the tumors collected from mice treated with different conditions at day 14. d) Average weights and volume of tumors collected from mice treated with different conditions at day 14. e) Body weight changes of mice treated with different conditions within 14 days. f) TUNEL confocal fluorescence images of dissected tumor tissue after 120 h treatment with PBS (I), CDs (II) SA Mn‐CDs (III). g) H&E‐stained tumor sections from mice with different treatments. (I) PBS, (II) CDs, and (III) SA Mn‐CDs. h) •OH intensity quantified from 8 random fields of view selected from each group. The scale bar is 100 µm. Statistical analysis was performed using the unpaired Student's t‐test (^***^ *p* < 0.001, ^****^ *p* < 0.0001).

According to the treatment scheme shown in **Figure** **6**a, the in vivo therapeutic efficacy of SA Mn‐CDs in the mouse model of invasive 4T1 breast cancer was further investigated. The mice were randomly assigned to three groups (n = 4). The control group was injected with PBS, while the treatment group was injected with CDs and SA Mn‐CDs, respectively. As observed in Figure 6b and Figure S22 (Supporting Information), SA Mn‐CDs aqueous solution and mice injected with SA Mn‐CDs (1 day after injection) produced bright NIR fluorescence in tumor tissues under 680 nm excitation, demonstrating its potential for NIR imaging‐guided cancer treatment. All mice were executed at the end of treatment (day 14) and tumors were collected for further analysis. As observed in Figure 6c, the groups treated with PBS and CDs exhibited a rapid growth of tumors. In contrast, the SA Mn‐CDs treatment group exhibited a notable reduction in tumor growth, suggesting that nanozymes can effectively inhibit tumor growth. As demonstrated in Figure 6d, the SA Mn‐CDs treatment group exhibited a notable reduction in both tumor volume and mass in comparison to the PBS and CDs treatment groups. The systemic toxicity of SA Mn‐CDs and CDs was evaluated through the measurement of body weight over the course of the treatment period. The growth curves of the mice in the treatment group demonstrated comparable weight fluctuations to those of the control mice, indicating that SA Mn‐CDs and CDs had no adverse effects on the health of the mice (Figure 6e). TUNEL staining was used to detect apoptosis in the main organs of mice treated with PBS/CDs/SA Mn‐CDs respectively. As illustrated in Figure 6f, the apoptosis of tumor cells was augmented in the SA Mn‐CDs‐treated cohort. H&E staining experiments revealed that a notable degree of tissue damage and loss of nuclei in the tissue sections of mice that had been injected with SA Mn‐CDs in comparison to the control and CDs treatment groups. This phenomenon may be associated with the generation of reactive oxygen species (ROS) and the mediation of apoptosis in the presence of SA Mn‐CDs. ROS staining of frozen tumor tissues was conducted using the fluorescent probe DCFH‐DA. The results demonstrated that mice treated with SA Mn‐CDs exhibited pronounced green fluorescence, indicative of a Fenton‐like reaction at the tumor site, which led to the inhibition of tumor proliferation (Figure 6g; Figure S23, Supporting Information).

## 3 Conclusion

In summary, we reported the simple preparation of SA Mn‐CDs on CDs carrier in Mn‐N_2_ configuration. SA Mn‐CDs showed good dispersibility of different solvents and excellent biological safety, and showed deeper (34 µm) two‐photon fluorescence imaging ability and exclusive function (Pearson's correlation coefficient: 0.752) of targeting lysosomes at the cellular level. This further promoted the high‐efficiency POD‐like activity (SA = 0.64 U mg^−1^) produced by a Fenton‐like reaction mediated by SA Mn‐CDs, and showed excellent ability to kill tumor cells and shrink tumor tissue in vitro and in vivo respectively. This work proves the great potential of monoatomic Mn nanozyme in NIR‐oriented tumor catalytic therapy.

## 4 Experimental Section

### Synthesis of SA Mn‐CDs and CDs

MnCl_2_•4H_2_O (40 mg) was dissolved in deionized water containing EDTA (20 mL, 50 mm). After 10 min of sonication, EDA (100 µL) was added to the above solution. The precursor solution was transferred to a Teflon‐lined stainless‐steel reactor and heated in an oven at 180 °C for 20 h. After cooling to room temperature, the product was centrifuged to remove the bottom sediment. The supernatant was collected with a dialysis bag (cutoff M_n_: 3000 D) and dialyzed for two days. Finally, SA Mn‐CDs were obtained by freeze‐drying under a vacuum. The pure CDs were prepared in accordance with the same procedure as the SA Mn‐CDs with the exception of the addition of MnCl_2_•4H_2_O.

### Characterization

The Cu Kα radiation (λ = 1.54178 Å) was used to obtain the X‐ray diffraction (XRD) pattern of SA Mn‐CDs and CDs using an X‐ray diffractometer (Rigaku D/MAX‐γA, Japan). Tecnai TF‐20 transmission electron microscope was used to capture high‐resolution TEM (HRTEM) images as well as transmission electron microscopy (TEM). Al Kα radiation was used to conduct X‐ray photoelectron spectroscopy (XPS) studies on an ESCALAB 250 X‐ray photoelectron spectrometer. Riley UV‐2601 spectrometer was used to record the room temperature UV–vis spectra of SA Mn‐CDs. At room temperature, the zeta potential was measured using a dynamic light scattering device (DLS, nano‐2s90, Malvern). A Horiba Jobin YvonT64000 micro‐Raman instrument was utilized to perform Raman scattering measurements with a torus 532 laser (λ = 532 nm) as the excitation source. A Nicolet Instruments Co.is10 FT‐IR spectrometer was used to record the Fourier transform infrared (FT‐IR) spectra. A PerkinElmer IVIS Lumina Series III imaging equipment was used to measure the fluorescence intensity. The Bruker EMX plus 10/12 was utilized for experiments using the electron spin resonance (ESR) spectrometer. The Mn *k*‐edge X‐ray absorption near edge structure (XANES) study was conducted at Beijing Synchrotron Radiation Facility's (1W1B) end station (BSRF).

### Evaluation of the Ability of SA Mn‐CDs or CDs to Generate •OH

20 µL of SA Mn‐CDs (1 mg mL^−1^) or CDs (1 mg mL^−1^), 150 µL of TMB (10 mg mL^−1^) and different volumes of 30% H_2_O_2_ were mixed and added to 3 mL of HAc‐NaAc buffer (pH 6.2). Using UV–vis spectroscopy, the absorbance of oxidized TMB at 651 nm was measured on a regular basis. Data fitting was used to determine the maximum velocities (V_max_) and Michaelis–Menten constants (K_m_) of SA Mn‐CDs.

### Detection of •OH Radical Generation by SA Mn‐CDs or CDs

The ESR apparatus was filled with capillary tubes that held either SA Mn‐CDs or CDs or 20 µL of pure water. Hydroxyl radical spin adducts (DMPO/•OH) were used to detect the characteristic peaks (1:2:2:1) of SA Mn‐CDs catalyzed production of •OH from H_2_O_2_ at different pH values. The ESR signal intensity provided a quantitative estimate of the amount of •OH.

### In Vitro Cell Viability of SA Mn‐CDs

In 96‐well plates, 4T1 cells were seeded at a density of 3.5 × 10^3^ cells/well (100 µL well^−1^) and allowed to incubate for the entire night. Subsequently, the cells were cultured for 24 h at varying pH values (6.2 and 7.4) and concentrations (0, 25, 50, and 100 µg mL^−1^) in PBS and SA Mn‐CDs solutions. Using a microplate reader (MD‐spectra EMaxComaxplus, Molecular Devices, Sunnyvale, America), the fluorescence intensity was measured and the cell viability was assessed using the CCK‐8 assay.

### Assessment of Intracellular ROS Levels

To find intracellular ROS generation, 2,7‐dichlorohydrofluorescein diacetate (DCFH‐DA) was employed as a ROS probe. In 12‐well plates, 4T1 cells were injected and left to culture for the entire night. 4T1 cells were treated with several doses (0, 25, 50, and 100 µg mL^−1^) of SA Mn‐CDs solutions and allowed to incubate for a full day. After treating 4T1 cells with SA Mn‐CDs for 20 min, DCFH‐DA was added. The cells were then rinsed three times with PBS. Ultimately, fluorescence pictures were captured at λ_ex_ = 488 nm using a Shanghai Leica DFC310 FX fluorescent microscope.

### Calcein‐AM/Prodium Iodide (PI) Double Staining of Live/Dead Cells

In RPMI‐1640 media, 4T1 cells were inoculated onto glass covers of 12‐well plates and incubated for the entire night at 37 °C. Following that, the cells were cultured for 24 h at various concentrations (0, 25, 50, and 100 µg mL^−1^) and pH values (6.2 and 7.4) using PBS and SA Mn‐CDs solutions. A LEICA DFC310 FX fluorescent microscope was used to capture fluorescence images of the treated cells after they had been stained for 15 min with calcein‐AM (green, living cells) and PI (red, dead cells). The two excitation wavelengths were 490 and 545 nm.

### Biosafety, Haematological Analysis, Biodistribution and Histopathological Evaluation

Beijing Witte River Laboratory Animal Science and Technology Co Ltd (Beijing, China) provided the four‐week‐old female BALB/c (nu/nu) mice that was bought. The Hefei Institute of Physical Sciences' Guidelines for the Care and Use of Laboratory Animals were followed in all animal experimentation procedures, and the Ethics and Humanities Committee of the Hefei Institute of Physical Sciences gave its approval (approval no. SWYX‐DW‐2022‐32, Chinese Academy of Sciences).

After 0.5 mL of diluted erythrocytes were combined with PBS, water, and SA Mn‐CDs solutions (12.5, 25, 50, and 100 µg mL^−1^), they were incubated for 4 h at 37 °C and centrifuged for 10 min at 3000 rpm. Water‐ and PBS‐treated erythrocytes were employed as positive and negative controls, respectively. After gathering the supernatant, the absorbance at 540 nm was measured. Hemolysis rate: (A_sample_‐A_negative_)/(A_positive_‐A_negative_) × 100% is the hemolysis rate (%). At 540 nm, sample, A_positive_, and A_negative_ represented the absorbance intensities of various SA Mn‐CD concentrations, water, and PBS.

Female BALB/c mice (n = 3) received intravenous injections of either PBS solution (control) or 200 µL of SA Mn‐CDs distributed in PBS at a concentration of 1 mg mL^−1^. 200 µL of blood with 0.15% (M/V) EDTA‐K_2_∙2H_2_O (EDTA double potassium salt, standard anticoagulant) was drawn for routine and blood biochemical testing after 24, 72, and 120 h of treatment, respectively. Serum specimens were obtained for blood biochemical analyses after whole blood was centrifuged.

To examine the biological distribution of SA Mn‐CDs in mice, main organs (heart, liver, spleen, lung, and kidney) and tumor tissues were extracted at various intervals after injecting PBS/SA Mn‐CDs into female mice via the tail vein. The IVIS imaging system was used to get the biological deposition of SA Mn‐CDs in various organs and tumor tissues, as well as the major organs and tumor tissues treated at different times. On day five, the mice were put to death and their major organs—the heart, liver, spleen, lungs, and kidneys—were removed after receiving injections of SA Mn‐CDs solution and PBS. The organs were sectioned for hematoxylin‐eosin (H&E) staining after being soaked in 4% paraformaldehyde for 24 h, followed by dehydration and paraffin embedding.

### In Vivo Therapeutic Effect of SA Mn‐CDs and ROS Detection in Tumor Site

4T1 cell line hormonal mice were randomly divided into three groups (n = 4 for each group). When the tumor volume reached ≈50 mm^3^, PBS, CDs (200 µL, 1 mg mL^−1^), and SA Mn‐CDs (200 µL, 1 mg mL^−1^) were injected intravenously on days 0, 2, and 4, respectively. Every two days, measurements of the tumor's length (L), breadth (W), and body weight were taken. V = (L × W^2^)/2 was used to compute the tumor volume. The relative tumor volume was measured as V/V_0_ (V_0_: initial volume before treatment). The mice were put to death after 14 days. H&E staining and photographing of tumor samples were conducted. Furthermore, the tumor tissues from mice treated with PBS or CDs or SA Mn‐CDs were cut into frozen sections with a thickness of 10 microns, stained for 30 min with a working solution, and then three times with PBS. Ultimately, the fluorescence pictures of the tumor slices were examined right away at λ_ex_ = 488 nm using a fluorescent microscope (LEICA DFC310 FX, Germany). After combining DCFH‐DA (5 µL) with NaOH (10 µL, 10 mm) for 30 min, PBS (1 mL) and HCl (10 µL, 10 mm) were added one after the other to create the working solution. This practical solution was put to use right away after becoming ready.

## Conflict of Interest

The authors declare no conflict of interest.

## Supporting information



Supporting Information

## Data Availability

The data that support the findings of this study are available from the corresponding author upon reasonable request.

## References

[1] a) S. Ji , B. Jiang , H. Hao , Y. Chen , J. Dong , Y. Mao , Z. Zhang , R. Gao , W. Chen , R. Zhang , Q. Liang , H. Li , S. Liu , Y. Wang , Q. Zhang , L. Gu , D. Duan , M. Liang , D. Wang , X. Yan , Y. Li , Nat. Catal. 2021, 4, 407;

[2] a) K. Kim , J. Lee , O. K. Park , J. Kim , J. Kim , D. Lee , V. K. Paidi , E. Jung , H. S. Lee , B. Lee , C. W. Lee , W. Ko , K. Lee , Y. Jung , C. Lee , N. Lee , S. Back , S. H. Choi , T. Hyeon , Adv. Mater. 2023, 35, 2207666; 10.1002/adma.202207666 36854306

[3] a) X. Wang , Z. Chen , X. Zhao , T. Yao , W. Chen , R. You , C. Zhao , G. Wu , J. Wang , W. Huang , J. Yang , X. Hong , S. Wei , Y. Wu , Y. Li , Angew. Chem. Int. Ed. 2018, 57, 1944; 10.1002/anie.201712451 29266615

[4] a) Z. Li , F. Liu , C. Chen , Y. Jiang , P. Ni , N. Song , Y. Hu , S. Xi , M. Liang , Y. Lu , Nano Lett. 2023, 23, 1505; 36734468 10.1021/acs.nanolett.2c04944

[5] a) X. Zhu , J. Wu , R. Liu , H. Xiang , W. Zhang , Q. Chang , S. Wang , R. Jiang , F. Zhao , Q. Li , L. Huang , L. Yan , Y. Zhao , ACS Nano 2022, 16, 18849; 36278792 10.1021/acsnano.2c07691

[6] a) S. Li , L. Li , H. Tu , H. Zhang , D. S. Silvester , C. E. Banks , G. Zou , H. Hou , X. Ji , Mater. Today 2021, 51, 188;

[7] a) H. He , Z. Fei , T. Guo , Y. Hou , D. Li , K. Wang , F. Ren , K. Fan , D. Zhou , C. Xie , C. Wang , X. Lu , Biomaterials 2022, 280, 121272; 34864428 10.1016/j.biomaterials.2021.121272

[8] a) D. Song , H. Guo , K. Huang , H. Zhang , J. Chen , L. Wang , C. Lian , Y. Wang , Mater. Today 2022, 54, 42;

[9] a) C. Peng , R. Pang , J. Li , E. Wang , Adv. Mater. 2024, 36, 2211724; 10.1002/adma.202211724 36773312

[10] a) Y. Cai , J. Fu , Y. Zhou , Y.‐C. Chang , Q. Min , J.‐J. Zhu , Y. Lin , W. Zhu , Nat. Commun. 2021, 12, 586; 33500393 10.1038/s41467-020-20769-x PMC7838205

[11] Y. Zhang , Q. Jia , F. Nan , J. Wang , K. Liang , J. Li , X. Xue , H. Ren , W. Liu , J. Ge , P. Wang , Biomaterials 2023, 293, 121953. 36521428 10.1016/j.biomaterials.2022.121953

[12] Y. Guo , Y. Sun , X. Geng , J. Wang , J. Hu , R.‐B. Song , R. Yang , L. Qu , Z. Li , Adv. Funct. Mater. 2024, 34, 2401744.

[13] S. Zhang , Y. Zha , Y. Ye , K. Li , Y. Lin , L. Zheng , G. Wang , Y. Zhang , H. Yin , T. Shi , H. Zhang , Nano‐Micro Lett. 2024, 16, 9. 10.1007/s40820-023-01217-z PMC10628069 37932531

[14] Y. Zhu , W. Wang , J. Cheng , Y. Qu , Y. Dai , M. Liu , J. Yu , C. Wang , H. Wang , S. Wang , C. Zhao , Y. Wu , Y. Liu , Angew. Chem. Int. Ed. 2021, 60, 9480. 10.1002/anie.202017152 33543825

[15] W. Xu , H. Li , X. Zhang , T.‐Y. Chen , H. Yang , H. Min , X. Shen , H.‐Y. Chen , J. Wang , Adv. Funct. Mater. 2024, 34, 2309509.

[16] Y. Qian , J. Zou , J. Zhang , X. Wang , X. Meng , Y. Lin , W. Lin , M. Zhang , H. Wang , Chem. Eng. J. 2024, 490, 151867.

[17] J. Wang , J. Li , Z. Yu , X. Zhu , J. Yu , Z. Wu , S. Wang , H. Zhou , Anal. Chem. 2022, 94, 14029. 36173258 10.1021/acs.analchem.2c03408

[18] X. Wei , Y. Li , H. Chen , R. Gao , P. Ning , Y. Wang , W. Huang , E. Chen , L. Fang , X. Guo , C. Lv , Y. Cheng , Adv. Sci. 2024, 11, 2302093. 10.1002/advs.202302093 PMC10916606 38095513

[19] B. Jiang , D. Duan , L. Gao , M. Zhou , K. Fan , Y. Tang , J. Xi , Y. Bi , Z. Tong , G. F. Gao , N. Xie , A. Tango , G. Nie , M. Liang , X. Yan , Nat. Protoc. 2018, 13, 1506. 29967547 10.1038/s41596-018-0001-1

