# Supplementary material for: Low‐Coordination Configuration Single‐Atom Manganese Nanozymes for NIR‐Imaging‐Oriented Efficient Catalytic Oncotherapy
Source: Adv Sci (Weinh). 2025 Mar 17;12(18):2502664. doi: 10.1002/advs.202502664 (PMC12079523; doi:10.1002/advs.202502664)
Supplement: Supplementary file 1 — Supporting Information [file ADVS-12-2502664-s001.docx]

Supporting Information

**Low-coordination Configuration Single-Atom Manganese Nanozymes for NIR-Imaging-Oriented Efficient Catalytic Oncotherapy**

*Peiwei Jin, Dandan Wang, Yijun Lu, Yong Qian^*^, Chengyang Fang, Xiaoxiao Zhang, Changwei Li,^*^ Junchao Qian,^*^ Songnan Qu,^*^ and Hui Wang^*^*

**Supplementary Figures**

**
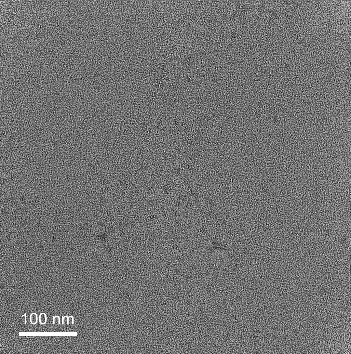
**

**Figure S1.** TEM image of CDs.





**Figure S2.** The zeta potential of SA Mn-CDs aqueous solution.


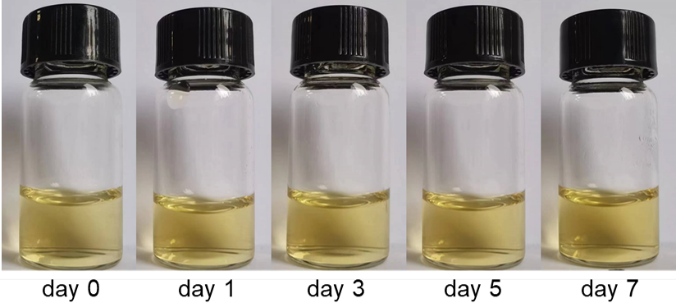


**Figure S3.** Digital photographs of SA Mn-CDs in aqueous solution for different days.


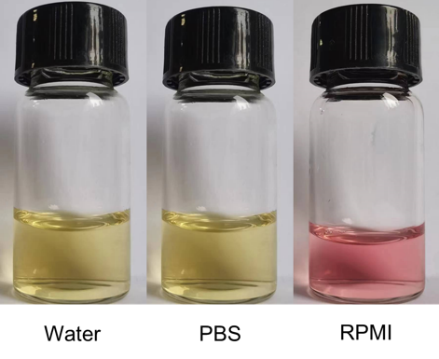


**Figure S4.** Dispersion photos of SA Mn-CDs in water, PBS and RPMI respectively.


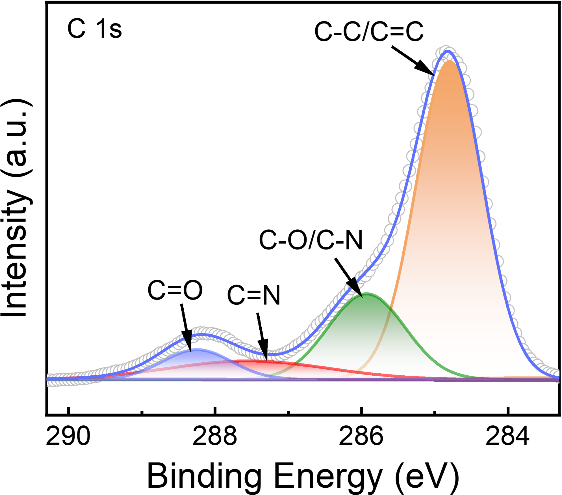


**Figure S5.** C 1s XPS of SA Mn-CDs.


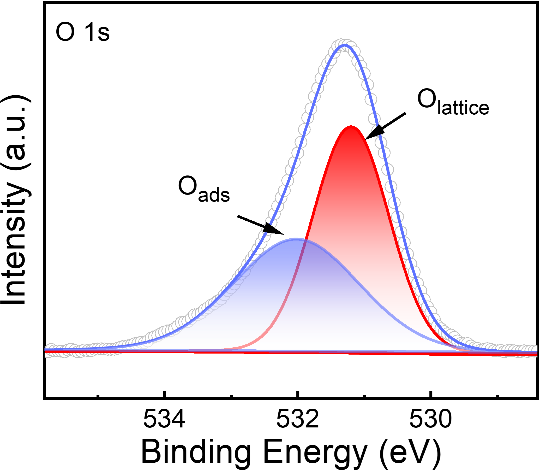


**Figure S6.** O 1s XPS of SA Mn-CDs.





**Figure S7.** The FT-EXAFS k space fitting curve of SA Mn-CDs.


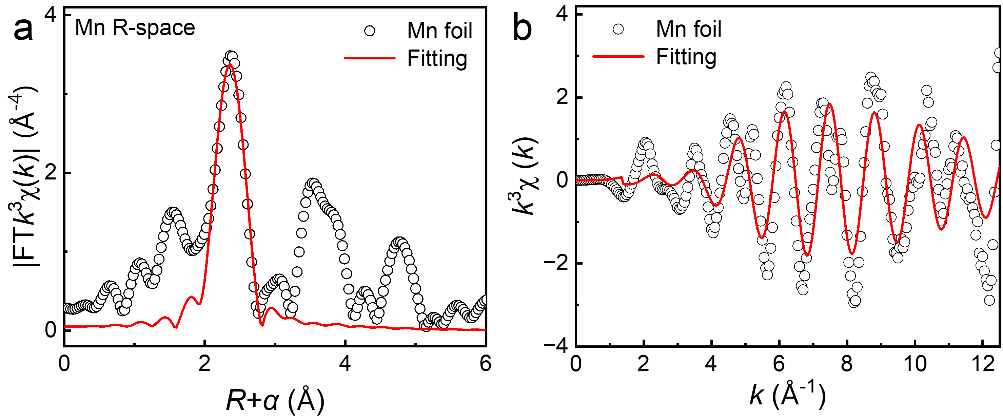


**Figure S8.** The FT-EXAFS R space fitting curve (a) and k space fitting curve (b) of Mn foil.


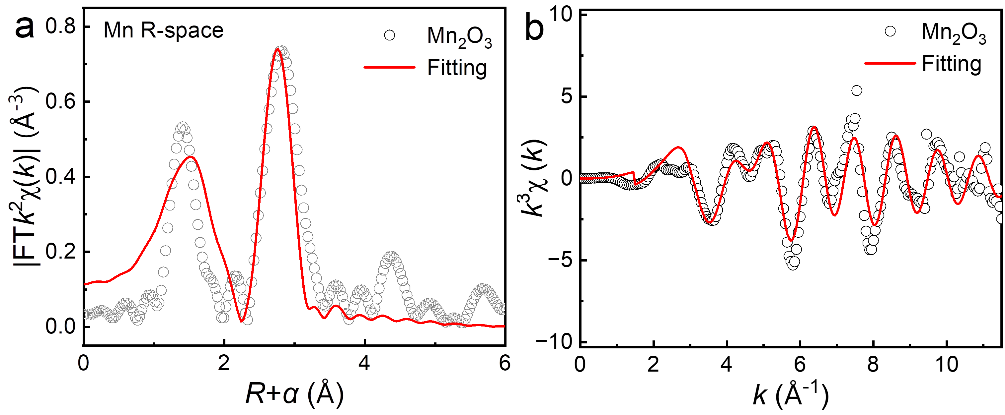


**Figure S9.** The FT-EXAFS R space fitting curve (a) and k space fitting curve (b) of Mn_2_O_3_ reference.





**Figure S10.** Relative fluorescence change of SA Mn-CDs irradiated by 365 nm for 60 min.


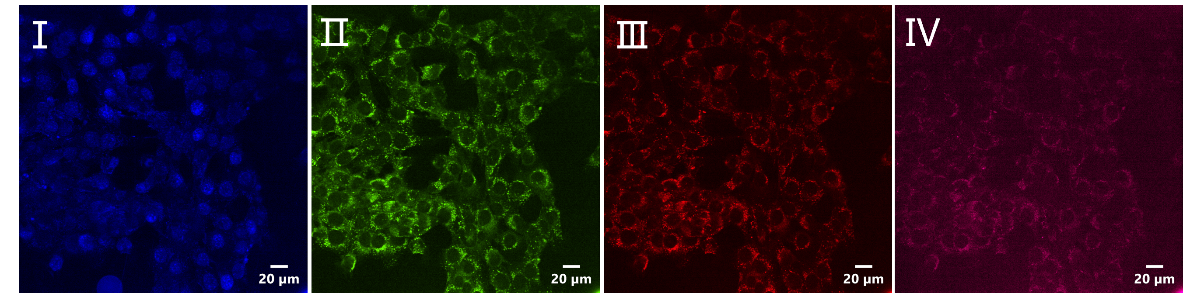


**Figure S11.** Confocal scanning imaging of 4T1 cells treated by SA Mn-CDs with an excitation wavelength of (Ⅰ) 405, (Ⅱ) 488, (Ⅲ) 561, and (Ⅳ) 640 nm, respectively.


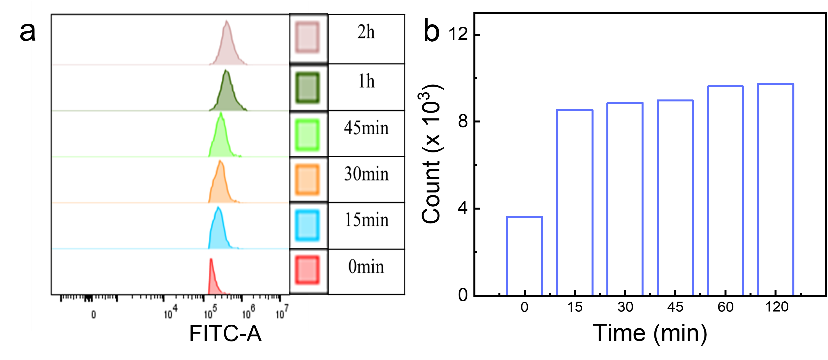


**Figure S12.** (a) Flow cytometry analysis and (b) corresponding quantitative fluorescence intensities for treatment with SA Mn-CDs at different times.


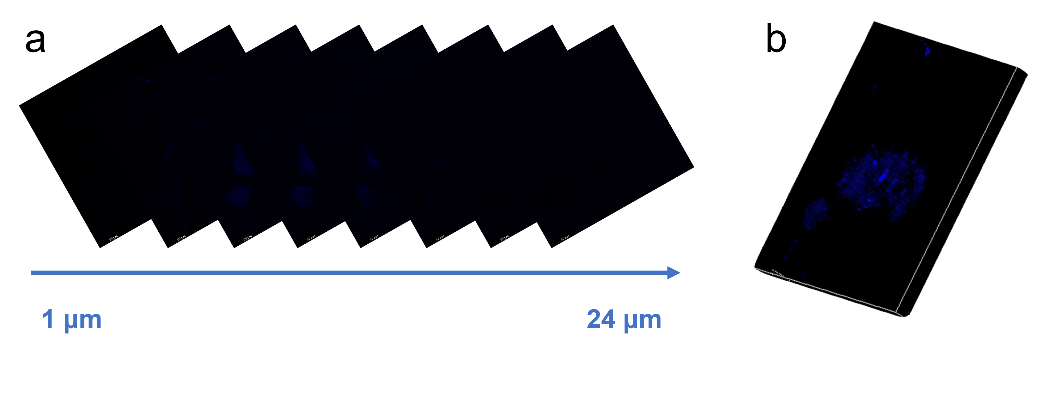


**Figure S13.** (a) The one-photon fluorescent images of fixed tumor cells stained with SA Mn-CDs at different penetration depths along the z-axis and (b) reconstructed 3D fluorescent images.





**Figure S14.** Time-dependent absorbance changes at 652 nm by adding various concentrations of H_2_O_2_ in the presence of the SA Mn-CDs.





**Figure S15.** Time-dependent absorbance changes at 652 nm by adding various concentrations of TMB in the presence of the SA Mn-CDs.





**Figure S16.** Michaelis-Menten fitting curves of •OH generation rate as function of TMB concentration in the presence of SA Mn-CDs and H_2_O_2_.





**Figure S17.** Lineweaver-Burke fitting curve of •OH generation rate as function of TMB concentration in the presence of SA Mn-CDs and H_2_O_2_.





**Figure S18.** Quantitative analysis of intracellular ROS levels based on Fig. 4i.


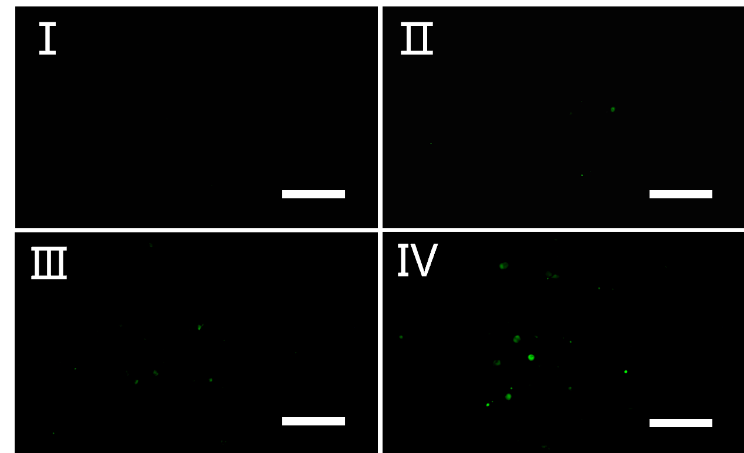


**Figure S19.** ROS staining of 4T1 cells incubated with different concentrations of SA Mn-CDs at pH 7.4. (I) 0, (II) 25, (III) 50 and (IV) 100 μg mL^−1^. The scale bar is 100 μm.


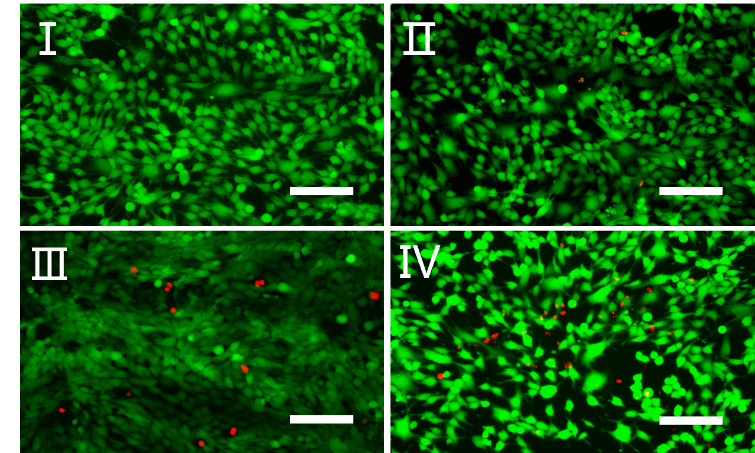


**Figure S20.** Dead/live staining of 4T1 cells treated with various concentrations of SA Mn-CDs at pH 7.4. (I) 0, (II) 25, (III) 50 and (IV) 100 μg mL^−1^. The scale bar is 100 μm.


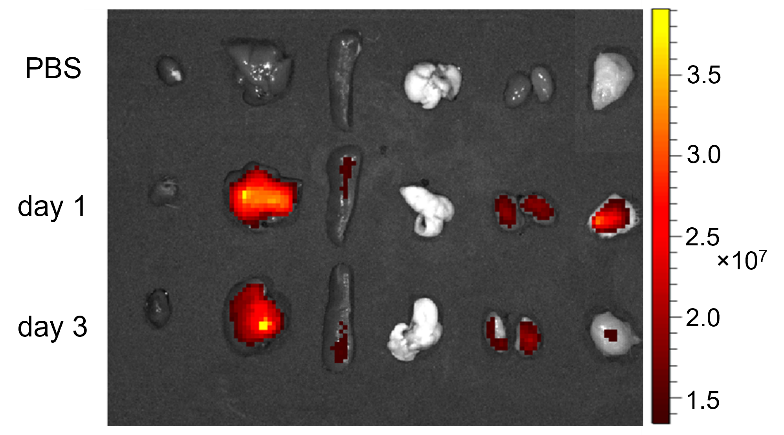


**Figure S21.** Fluorescence intensity of different main organs and tumor tissues of tumor-bearing mice treated with SA Mn-CDs at different time points under IVIS imaging system. From left to right are heart, liver, spleen, lung, kidney and tumor.


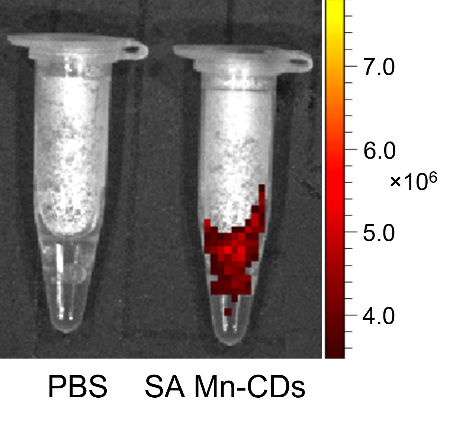


**Figure S22.** Fluorescence photographs of PBS and SA Mn-CDs solutions under excitation at 680 nm.


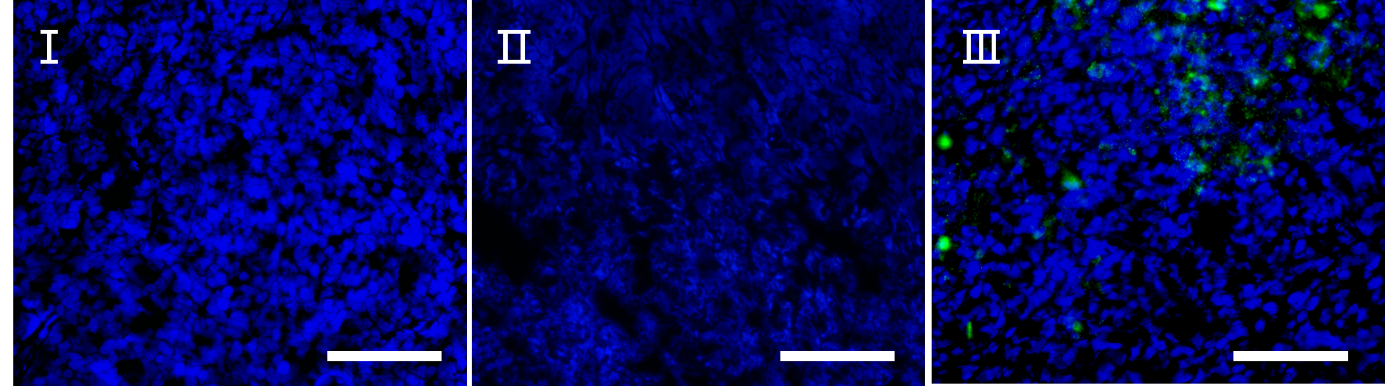


**Figure S23.** DCFH-DA-stained sections of tumor from mice treated with different conditions. (I) PBS, (II) CDs, (Ⅲ) SA Mn-CDs. The scale bar is 200 µm.

**Table S1.** EXAFS Fitting Parameters at the Mn K-edge for Various Samples.

| **Sample** | **Path** | **CN** | **R (Å)** | **σ^2^ (10^-2^Å^2^)** | **ΔE_0_ (eV)** | **R factor** |
| --- | --- | --- | --- | --- | --- | --- |
| **SA Mn-CDs** | Mn-N | 2.2±0.1 | 2.21±0.01 | 0.7±0.2 | 2±1 | 0.02 |
| **Mn foil** | Mn-Mn | 12 | 2.73±0.02 | 0.4±0.2 | 7±2 | 0.012 |
| **Mn_2_O_3_** | Mn-O | 6 | 1.96±0.01 | 0.7±0.3 | 8±2 | 0.018 |
|  | Mn-Mn | 6 | 1.96±0.02 | 0.7±0.3 | 8±2 | 0.018 |

CN is the coordination number; R(Å) is interatomic distance (the bond length between central atoms and surrounding coordination atoms); σ^2^(Å^2^) is Debye-Waller factor (a measure of thermal and static disorder in absorber-scatterer distances); ΔE_0_(eV) is edge-energy shift (the difference between the zero kinetic energy value of the sample and that of the theoretical model). R factor is used to value the goodness of the fitting. S_0_^2^ was fixed to 0.9. The data range used for data fitting in k-space (∆k) and R-space (∆R) are 3.0-12.4 Å^−1^ and 1.0-3.0 Å, respectively.
